# Associations between body height and cardiovascular risk factors in women and men: a population-based longitudinal study based on The Tromsø Study 1979–2016

**DOI:** 10.1136/bmjopen-2024-084109

**Published:** 2024-10-17

**Authors:** Sondre Haakonson Arntsen, Tom Wilsgaard, Kristin Benjaminsen Borch, Inger Njolstad, Anne Helen Hansen

**Affiliations:** 1 Department of Community Medicine, UiT The Arctic University of Norway, Tromso, Norway; 2 University Hospital of North Norway, Tromso, Norway

**Keywords:** Blood Pressure, Observational Study, Public Health, Social Medicine, Cardiac Epidemiology

## Abstract

**Abstract:**

**Objectives:**

Investigate associations between body height and cardiovascular disease risk factors at several time points in women and men across educational levels in Norway.

**Design:**

Population-based longitudinal study.

**Setting:**

The Tromsø Study, a population-based study with six surveys conducted between 1979 and 2016 in the municipality of Tromsø, Norway.

**Primary and Secondary Outcome Measures:**

Body height, systolic blood pressure, diastolic blood pressure, serum total cholesterol, high-density lipoprotein (HDL) cholesterol, triglycerides and self-reported educational level.

**Participants:**

23 512 women and men (49.6% women), aged 30–49 years at first participation in The Tromsø Study. Participants who attended more than one survey contributed with repeated measurements for blood pressure and lipids.

Blood pressure and lipid values were used as dependent variables in sex specific age-adjusted linear mixed models. Body height at first participation was the independent variable, while survey time point and educational level were used as covariates.

**Results:**

Overall effect models showed inverse associations between body height and systolic blood pressure (reg. coefficients: −0.88 (95% CI –1.1, −0.6)), diastolic blood pressure (−0.41 (95% CI –0.6, –0.3)), serum total cholesterol (−0.12 (95% CI –0.1, –0.1)) and triglycerides (−0.06 (95% CI –0.1, –0.0)) in women. Inverse associations between body height and lipid variables were also observed in men (serum total cholesterol: −0.12 (95% CI −0.1, –0.1) triglycerides −0.05 (95% CI –0.1, –0.0)). Regression coefficients for associations between body height and cardiovascular risk factors varied across surveys. Overall, there were no associations between body height and cardiovascular risk factors based on educational level and survey.

**Conclusion:**

The overall effect models support previous findings of inverse associations between body height and cardiovascular risk factors in women, and inverse associations between body height and lipids in men. Our study showed varied degrees of associations between body height and cardiovascular risk factors at different time points in Norway.

**STRENGTHS AND LIMITATIONS OF THIS STUDY**

- The main strength of our study is the investigation of associations between body height and cardiovascular risk factors at several time points, which to our knowledge has not been done previously in a Norwegian population.
- Another strength of the investigation of associations between body height and cardiovascular risk factors in a large population of women, which is currently under-represented in the literature.
- A third strength of our study is the collection of height measurements and repeated measurements of cardiovascular risk factors over four decades.
- Our study is limited by changes in equipment used for blood pressure measurements, differing units of measurements for height and education between surveys, multiple testing, which increases the chance of type I error and a lack of information on the confounding variables of parental socioeconomic status and early year living conditions, which may influence body height, educational level and cardiovascular risk factors.

## Introduction

 The inverse relationship between body height and cardiovascular disease is well established.^1^,^8^ Concurrently, differences in mean body height between socioeconomic status (SES) groups based on attained educational level have been observed, with taller women and men in groups with tertiary education as compared with women and men without tertiary education.^9^,^12^ On a similar note, differences between SES groups regarding traditional cardiovascular risk factors have been reported in several European countries, with higher systolic and diastolic blood pressure and blood lipid levels in groups with low educational levels.^13^,^16^ While there have been several studies on body height and cardiovascular disease, studies focusing on associations between body height and cardiovascular risk factors are less common.

A 2021 literature review found tall stature to be associated with lower blood pressure.^17^ Sex differences in the associations between body height and blood pressure have been reported in several studies, suggesting that the advantage of being tall in comparison to mean population height is more pronounced in women than in men.^18^,^20^ Low body height in relation to mean population height has been associated with an increased risk of elevated total cholesterol and elevated triglycerides and/or reduced high-density lipoprotein (HDL) cholesterol in Asian and European populations.^21^,^24^

Currently, associations between body height and cardiovascular risk factors are less investigated among women compared with men, and most studies on this subject are cross-sectional studies.^20 21 25 26^ To the best of our knowledge, there have been no studies investigating the association between body height and cardiovascular risk factors across educational levels at several time points in a Norwegian population.

The aim of our study was to investigate the association between body height and cardiovascular risk factors at several time points in women and men across educational levels in Norway.

## Methods

### Design and data

In this population-based longitudinal study, we used previously collected data from The Tromsø Study, a population-based health study with seven surveys conducted between 1974 and 2016 in the municipality of Tromsø, Norway. The Tromsø Study started in the 1970s as a combined screening and research study in response to the high rates of cardiovascular morbidity and mortality in northern regions of Norway compared with the rest of the country.^27^ Women and men residing in Tromsø have been invited to attend at regular intervals since 1974. Between 1979 and 2016, a total of 59 722 residents were invited to participate in The Tromsø Study, and 44 489 women and men participated in one or more surveys.

In our study, we used data from Tromsø2–7 (1979–2016), since Tromsø1 (1974) invited men only. Each Tromsø Study survey can be considered its own cross-sectional study, with the possibility of participants being invited to and attending multiple surveys allowing studies with repeated measurements within the wider population of The Tromsø Study.

Data used in in our study were collected from clinical measurements, laboratory measurements and questionnaires, which is described in more detail in the variables section. Data on sex and age were derived from the national 11-digit unique personal identification number provided by the Norwegian National Population Registry. The cohort profile and data collection in The Tromsø Study is described in detail elsewhere.^28 29^ Our study was approved by the Norwegian Regional Committee for Medical and Health Research Ethics North (REC North).

### Patients and public involvement

There was no patient or public involvement in this particular study.

### Participants

**Figure 1:**
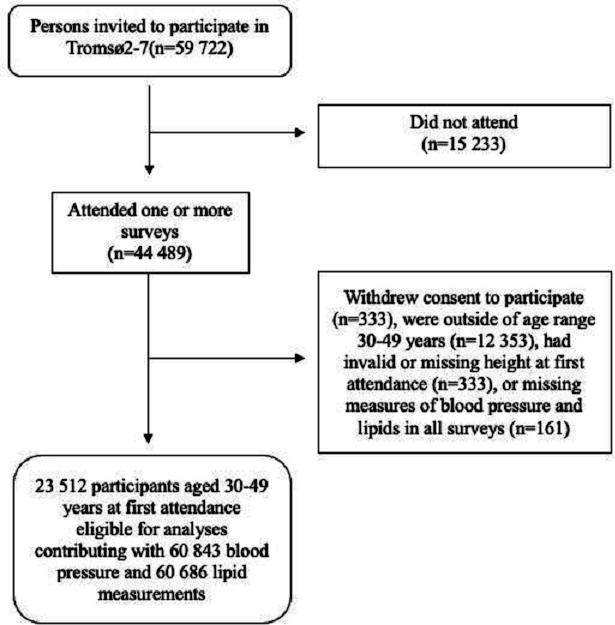
Flow chart of study participants.

Inclusions and exclusions of participants are presented in figure 1. We included participants aged 30–49 years. Participants who were younger than 30 years at first attendance to The Tromsø Study, were included at their first attendance past the age of 30 years, to ensure that more participants reached their highest educational level at the time of inclusion. We used the body height measurement from first attendance also for those who attended one or more later surveys. The age restriction of 49 years at first participation was chosen to ensure minimal height loss related to ageing.^30 31^ All participants contributed with at least one measurement of blood pressure and lipid variables and participants with more than one attendance provided repeated measurements.

We excluded those who withdrew after data collection (n=333), were outside of the age range of 30–49 years at first participation (n=20 150), had invalid height measurements (missing or height measured wearing shoes or headgear, n=333) or missing measures of cardiovascular risk factors (n=161).

The final study sample consisted of 23 512 participants (49.6% women), where 4331 participants (18.4%) attended two surveys, 2864 participants (12.2%) attended three surveys, 2507 participants (10.7%) attended four surveys, 2607 participants (11.1%) attended five surveys and 1885 participants (8.0%) attended all six surveys. The participants included in our study contributed with 60 843 observations for blood pressure variables and 60 686 observations for lipid variables.

### Variables

The dependent and independent variables were obtained from measurements conducted by trained research technicians. The dependent variables in our analyses were systolic blood pressure, diastolic blood pressure, serum total cholesterol, HDL cholesterol and triglycerides. Body height was the independent variable in our study. The adjustment variable in the main analyses was age, while overall effect models were adjusted for age and survey. Sex was used as a stratification variable, while survey and educational level were used as covariates.

The equipment used to measure blood pressure varied between survey time points. A mercury sphygmomanometer was used to manually measure blood pressure in Tromsø2, while two automatic devices were used in Tromsø3–7. The Dinamap Vital Signs Monitor (Critikon, Tampa, Florida, USA) was used in Tromsø3–5, while the Dinamap ProCare 300 (GE Medical Systems Information Technologies, Tampa, Florida, USA) was used in Tromsø6–7. However, the measurement procedure was similar across surveys. Blood pressure was measured two times in Tromsø2 and three times (Tromsø3–7) in a seated position after a short rest. We used the second measurement of blood pressure in Tromsø2, since it was the lowest reported value for systolic and diastolic blood pressure.

In Tromsø3–7, mean systolic blood pressure and diastolic blood pressure were calculated based on the second and third measurements. We used this variable in our study since it was the available blood pressure variable from Tromsø3–7 in our data set.

The lipid variables of serum total cholesterol, HDL cholesterol and triglyceride concentrations were obtained from non-fasting blood samples. All blood samples were analysed within 48 hours at The Department of Laboratory Medicine, University Hospital of North Norway.

Body height in The Tromsø Study was measured in a standing position wearing light clothing and no footwear, although measurement equipment and reporting of measured body height differed slightly between surveys. In Tromsø2–4, body height was measured to the closest 1 cm with a wall-mounted ruler. While, body height was measured to the closest 0.1 cm, using an automatic electronic scale, Jenix DS 102 stadiometer (Dong Sahn Jenix, Seoul, Korea) in Tromsø5–7. To account for the different reporting of height measurements between Tromsø2–4 and Tromsø5–7, we checked the data by browsing body height in participants with multiple measurements. We found all body height differences to be less than 1.0 cm between the reported measurement methods.

Data on attained educational level was obtained for questionnaires, where the questions for reporting educational level differed between surveys. Open response categories were used in Tromsø2, 3 and 5 with participants filling in the number of years of education they had completed.

In Tromsø4 and Tromsø6, participants were asked ‘What is the highest educational level you have completed?’ with five answering options (primary school, vocational school, upper secondary school, college/university education less than 4 years and college/university education 4 years or more). The same question was used in Tromsø7 with the answer options of vocational school and upper secondary school were merged into one response (upper secondary school).

In order to have one standardised variable for educational level across surveys, we decided to align our educational groups with the groups based on the questions in Tromsø7. Thus, we recoded years of education from Tromsø2–3 and 5 into the following levels (up to 9 years of education=primary education, 10–12 years=upper secondary education, 13–15 years=short tertiary education, 16+years=long tertiary education). The answer options of vocational school and upper secondary school from Tromsø4 and 6 was merged into one category (upper secondary education).

### Statistical analyses

Descriptive characteristics were reported as mean (SD) or number (per cent). Linear mixed models were used to assess the associations between blood pressure or lipid levels as dependent variables and body height as a continuous independent variable. Indicator variables of survey time points and educational level were added as covariates, along with all two-way and three-way cross-product terms between height and the indicator variables of survey time point and educational level. Based on the fitted model, we reported regression coefficients per 1 SD increase in body height for each survey time point and each level of education (1 SD=6.2 cm in women and 6.7 cm in men).

A random intercept on the participant level was included in the models to control for dependencies between repeated observations. In separate analyses, we estimated main effect regression coefficients for associations between body height and dependent variables adjusted for age and survey time point.

Normal distribution of all dependent variables, except for triglycerides was found adequate when assessed by histograms. The distribution of triglycerides was slightly skewed to the right. However, we chose not to transform triglycerides since regression models using large data sets are robust with respect to deviations from the normal distribution. The linearity assumption was found adequate when assessed by adding a quadratic and a cubic term of body height to models that included a linear term of height adjusted for age and survey time point. Outliers was checked using summary statistics for height, blood pressure and lipids.

Likelihood-ratio tests were used to assess the interaction between body height and educational level and body height and survey time points. P values from Wald tests were used to assess the difference in regression coefficients for body height between the first and the last time point (Tromsø2 vs Tromsø7). This test focused on the two-way interaction term between body height and the indicator variable of the last time point (Tromsø7), with the first time point (Tromsø2) set as the reference level in the previously specified linear mixed models.

Adjustments for age in our analyses were based on a directed acyclic graph (DAG) on the pathway between body height and individual cardiovascular risk factors (online supplemental figure S1), drawn using DAGitty.^32^ We used 95% CIs throughout the study, and a p value of<0.05 was considered statistically significant. All statistical analyses were sex-specific and conducted using Stata V.18.0 (StataCorp, College Station, Texas, USA).

## Results

Characteristics of study participants in each survey are presented in table 1. Women and men in Tromsø2 were younger and had a lower mean body height than participants in Tromsø7. The lowest mean systolic blood pressure in women was observed in Tromsø2, with higher mean values in each subsequent survey up to Tromsø7. In men, mean systolic blood pressure varied across the surveys with the highest mean measurement in Tromsø6 and the lowest in Tromsø2.

**Table 1:** Characteristics of study participants across surveys*, The Tromsø Study (1979–2016)

	Tromsø21979–1980	Tromsø31986–1987	Tromsø41994–1995	Tromsø52001	Tromsø62007–2008	Tromsø72015–2016
Women						
Number of observations	5046	5694	6594	2933	4274	6662
Age (years)	37.7 (5.9)	42.5 (6.9)	47.0 (9.3)	56.5 (12.1)	59.0 (11.5)	57.3 (13.1)
Body height (cm)	163.5 (6.1)	163.9 (6.2)	164.0 (6.3)	162.8 (6.3)	165.5 (6.2)	166.0 (6.4)
Systolic blood pressure (mm Hg)	122.5 (14.3)	125.0 (16.0)	130.1 (19.3)	135.1 (22.0)	136.0 (24.6)	127.6 (21.6)
Diastolic blood pressure (mm Hg)	79.6 (9.7)	76.9 (10.4)	76.8 (11.8)	78.4 (12.3)	75.2 (10.1)	72.6 (9.6)
Total serum cholesterol (mmol/L)	5.9 (1.2)	5.9 (1.3)	6.1 (1.3)	6.2 (1.2)	5.8 (1.1)	5.5 (1.1)
HDL cholesterol (mmol/L)	1.8 (0.4)	1.7 (0.4)	1.7 (0.4)	1.6 (0.4)	1.7 (0.4)	1.7 (0.5)
Triglycerides (mmol/L)	1.1 (0.6)	1.2 (0.7)	1.3 (0.8)	1.4 (0.8)	1.4 (1.0)	1.3 (0.7)
Primary education, n (%)	2649 (53.7)	2664 (47.5)	2673 (40.5)	1497 (51.1)	1693 (39.7)	1701 (25.6)
Upper secondary education, n (%)	1272 (25.8)	1449 (25.9)	1826 (27.7)	679 (23.2)	1165 (27.3)	1682 (25.3)
Short tertiary education, n (%)	560 (11.4)	693 (12.4)	968 (14.7)	345 (11.8)	612 (14.3)	1197 (18.0)
Long tertiary education, n (%)	449 (9.1)	799 (14.3)	1125 (17.1)	409 (14.0)	797 (18.7)	2066 (31.1)
Men						
Number of observations	5013	5687	6638	2237	3946	6221
Age (years)	37.9 (5.7)	42.5 (6.9)	46.9 (9.1)	56.7 (12.4)	59.0 (11.2)	57.6 (12.9)
Body height (cm)	176.8 (6.7)	177.0 (6.7)	177.2 (6.9)	176.1 (6.7)	179.2 (6.9)	179.4 (6.6)
Systolic blood pressure (mm Hg)	130.3 (13.6)	132.6 (14.3)	136.1 (16.2)	137.8 (18.8)	139.0 (20.3)	132.9 (18.2)
Diastolic blood pressure (mm Hg)	83.5 (10.1)	80.5 (10.5)	81.3 (11.2)	81.4 (11.5)	81.3 (10.2)	77.8 (9.7)
Total serum cholesterol (mmol/L)	6.2 (1.2)	6.1 (1.2)	6.2 (1.2)	6.0 (1.1)	5.5 (1.1)	5.3 (1.1)
HDL cholesterol (mmol/L)	1.5 (0.5)	1.4 (0.4)	1.4 (0.4)	1.3 (0.4)	1.4 (0.4)	1.4 (0.4)
Triglycerides (mmol/L)	1.4 (1.0)	1.7 (1.1)	1.8 (1.2)	1.7 (1.1)	1.6 (1.0)	1.7 (1.0)
Primary education, n (%)	2084 (42.8)	2161 (38.6)	2268 (34.2)	989 (44.3)	1311 (33.2)	1436 (23.1)
Upper secondary education, n (%)	1339 (27.5)	1548 (27.6)	1940 (29.2)	601 (26.9)	1137 (28.8)	1827 (29.4)
Short tertiary education, n (%)	670 (13.8)	840 (15.0)	1084 (16.3)	299 (13.4)	679 (17.2)	1207 (19.4)
Long tertiary education, n (%)	772 (15.9)	1050 (18.8)	1344 (20.3)	346 (15.5)	818 (20.7)	1742 (28.0)

* Values are mean (SD) or number (%). Primary education=primary school and secondary school/up to 9 years of education. Upper secondary education=upper secondary school/10–12 years of education. Short tertiary education=university/college <4 years. Long tertiary education=university/college >4 years.

HDLhigh-density lipoprotein

In contrast, mean diastolic blood pressure was lower in Tromsø7 compared with Tromsø2 in women and men. Mean serum total cholesterol and HDL cholesterol concentrations were lower in Tromsø7 compared with Tromsø2. Mean triglyceride concentration remained stable in all surveys. Overall, attained educational level differed between surveys with a higher percentage of observations in the tertiary education groups in Tromsø7 compared with Tromsø2.

### Educational level, body height and cardiovascular risk factors in women

Associations between body height and cardiovascular risk factors in women estimated by educational level and survey time points are presented in table 2. Overall, there were no associations between height and cardiovascular risk factors at survey time points or educational level. We observed no association between body height and systolic blood pressure in Tromsø2. However, significant inverse associations between body height and systolic blood pressure were found in the two lower educational groups in Tromsø7 (regression coefficients: primary education: −1.32 (95% CI –2.1, −0.7), upper secondary education −1.86 (–2.7, –1.1)).

**Table 2:** Linear mixed model regression coefficients (95% CI) for the association between body height and cardiovascular risk factors in women per 1 SD body height by educational level and survey in The Tromsø Study (1979–2016)

	Tromsø21979–1980	Tromsø31986–1987	Tromsø41994–1995	Tromsø52001	Tromsø62007–2008	Tromsø72015–2016	P value
Primary education (n=11 421)							
Systolic blood pressure (n=11 421)	0.17 (−0.5, 0.8)	−0.51 (−1.2, 0.1)	−0.33 (−1.0, 0.3)	−1.02 (−1.9, −0.2)	−1.28 (−2.1, −0.5)	−1.32 (−2.1, −0.5)	0.001
Diastolic blood pressure (n=11 421)	−0.07 (−0.5, 0.3)	−0.49 (−0.9, −0.1)	−0.33 (–0.7, 0.1)	−0.27 (−0.8, 0.2)	0.18 (−0.3, 0.6)	0.19 (−0.3, 0.6)	0.515
Serum total cholesterol (n=11 383)	−0.16 (−0.2, −0.1)	−0.16 (–0.2, −0.1)	−0.17 (–0.2, −0.1)	−0.04 (–0.1, 0.0)	0.06 (0.0, 0.1)	0.00 (−0.0, 0.1)	0.000
HDL cholesterol (n=11 374)	0.01 (−0.0, 0.0)	−0.00 (−0.0, 0.0)	0.00 (–0.0, 0.0)	0.01 (−0.0, 0.0)	0.03 (0.0, 0.0)	0.02 (0.0, 0.0)	0.137
Triglycerides (n=11 382)	−0.02 (−0.1, 0.0)	−0.04 (−0.1, −0.0)	−0.06 (−0.1, −0.0)	−0.04 (−0.1, –0.0)	−0.01 (−0.0, 0.0)	−0.05 (−0.1, −0.0)	0.445
Upper secondary education (n=7930)							
Systolic blood pressure (n=7930)	−0.41 (−1.4, 0.5)	−0.24 (−1.1, 0.6)	−0.77 (–1.5, –0.1)	−0.78 (–1.9, 0.4)	−0.88 (−1.7, −0.0)	−1.86 (−2.7, −1.1)	0.021
Diastolic blood pressure (n=7930)	−0.01 (−0.6, 0.6)	−0.42 (−0.9, 0.1)	−0.71 (−1.1, −0.3)	−0.91 (−1.6, −0.2)	0.37 (−0.1, 0.9)	−0.35 (−0.8, 0.1)	0.397
Serum total cholesterol (n=7920)	−0.14 (−0.2, −0.1)	−0.12 (−0.2, –0.1)	−0.13 (−0.2, –0.1)	−0.02 (−0.1, 0.1)	−0.03 (−0.1, 0.0)	−0.08 (−0.1, –0.0)	0.116
HDL cholesterol (n=7916)	0.01 (−0.0, 0.0)	−0.00 (−0.0, 0.0)	0.01 (−0.0, 0.0)	0.01 (−0.0, 0.0)	0.02 (0.0, 0.0)	0.02 (−0.0, 0.0)	0.761
Triglycerides (n=7919)	−0.04 (−0.1, −0.0)	−0.03 (−0.1, 0.0)	−0.05 (−0.1, −0.0)	−0.01 (−0.1, 0.0)	−0.06 (−0.1, −0.0)	−0.08 (−0.1, −0.0)	0.134
Short tertiary education (n=4170)							
Systolic blood pressure (n=4170)	0.36 (−1.1, 1.8)	−0.33 (−1.8, 0.8)	−1.00 (−2.0, −0.0)	−0.13 (−1.8, 1.5)	−1.05 (−2.3, 0.2)	−0.60 (−1.5, 0.4)	0.201
Diastolic blood pressure (n=4170)	−0.34 (–1.2, 0.5)	−0.66 (−1.3, 0.3)	−1.00 (–1.6,–0.4)	−0.03 (–1.0, 0.9)	0.40 (–0.4, 1.2)	0.21 (−0.4, 0.8)	0.309
Serum total cholesterol (n=4163)	−0.10 (−0.2, −0.0)	−0.16 (−0.2, –0.1)	−0.20 (–0.3, –0.1)	−0.05 (−0.2, 0.1)	−0.08 (−0.2, 0.0)	−0.06 (−0.1, 0.0)	0.355
HDL cholesterol (n=4161)	−0.01 (−0.0, 0.0)	0.01 (−0.0, 0.0)	−0.00 (–0.0, 0.0)	0.03 (–0.0, 0.1)	0.02 (–0.0, 0.1)	0.02 (−0.0, 0.0)	0.438
Triglycerides (n=4162)	−0.07 (−0.1, –0.0)	−0.05 (−0.1, 0.0)	−0.06 (−0.1, −0.0)	−0.03 (−0.1, 0.0)	−0.02 (−0.1, 0.0)	−0.04 (−0.1, 0.0)	0.183
Long tertiary education (n=6242)							
Systolic blood pressure (n=6242)	1.22 (−0.4, 2.8)	0.96 (−0.2, 2.1)	−0.04 (−0.9, 0.9)	−0.20 (−1.5, 1.1)	−1.48 (–2.5, −0.4)	−1.31 (−2.0, −0.7)	0.002
Diastolic blood pressure (n=6242)	0.80 (−0.1, 1.8)	−0.25 (−0.9, 0.4)	−0.57 (−1.1, −0.0)	−0.52 (−1.3, 0.3)	−0.40 (–1.0, 0.2)	−0.43 (−0.8, −0.0)	0.031
Serum total cholesterol (n=6228)	−0.05 (−0.2, 0.0)	−0.15 (−0.2, −0.1)	−0.17 (−0.2, −0.1)	−0.04 (−0,1 0.0)	−0.06 (−0.1, 0.0)	−0.04 (−0.0, 0.0)	0.918
HDL cholesterol (n=6226)	0.01 (−0.0, 0.0)	−0.00 (−0.0, 0.0)	0.00 (−0.0, 0.0)	0.03 (0.0, 0.1)	0.03 (0.0, 0.1)	0.04 (0.0, 0.1)	0.142
Triglycerides (n=6229)	−0.03 (−0.1, 0.0)	−0.05 (−0.1, −0.0)	−0.07 (−0.1, −0.0)	−0.05 (−0.1, 0.0)	−0.06 (−0.1, −0.0)	−0.09 (−0.1, −0.0)	0.037

Linear mixed models adjusted for age (n): number of observations pr cardiovascular risk factor, primary education=primary school and secondary school/up to 9 years of education. Upper secondary education=upper secondary school/10–12 years of education. Short tertiary education=university/college <4 years. Long tertiary education=university/college ≥4 years. P value=difference in regression coefficients between Tromsø2 and Tromsø7.

HDLhigh-density lipoprotein

There were no associations between body height and diastolic blood pressure, except in women with upper secondary or short tertiary education, where we observed significant inverse associations between body height and diastolic blood pressure in Tromsø4–5 (Tromsø4: upper secondary education: −0.71 (95% CI –1.1, –0.3), short tertiary education: −1.00 (95% CI –1.6, –0.4). Tromsø5: upper secondary education −0.91 (95% CI –1.6, –0.2)). Across all educational levels except for long tertiary education, we observed significant inverse associations between body height and serum total cholesterol in Tromsø2–4. However, the strength of these associations attenuated across survey time points, and there were no associations in Tromsø7.

There were no associations between body height and HDL cholesterol in Tromsø2–5. However, small but significant direct associations were found in all education groups except for the short tertiary education group in the Tromsø6–7. In the long tertiary education group, we observed significant inverse associations between body height and triglycerides at all time points, except for Tromsø2. Significant inverse associations were also observed in women with primary education, except for Tromsø6. No associations were observed in Tromsø5–7 in women with upper secondary or short tertiary education.

#### Educational level, body height and cardiovascular risk factors in men

Associations between body height and cardiovascular risk factors in men by educational level and survey time point are presented in table 3. Similar to our findings in women, there were no associations between body height and cardiovascular risk factors at most survey time points and there were no associations by survey time point and educational levels.

**Table 3:** Linear mixed model regression coefficients (95% CI) for the association between body height and cardiovascular risk factors in men per 1 SD body height by educational level and survey in The Tromsø Study (1979–2016)

	Tromsø21979–1980	Tromsø31986–1987	Tromsø41994–1995	Tromsø52001	Tromsø62007–2008	Tromsø72015–2016	P value
Primary education (n=8868)							
Systolic blood pressure (n=8868)	0.71 (0.0, 1.4)	0.64 (−0.0, 1.3)	−0.42 (−1.1, 0.3)	−1.04 (−2.0, −0.1)	−2.07 (−3.0, −1.2)	−0.43 (−1.2, 0.4)	0.051
Diastolic blood pressure (n=8868)	0.41 (−0.0, 0.9)	−0.05 (−0.5, 0.4)	−0.04 (–0.5, 0.4)	−0.33 (−0.9, 0.2)	−0.09 (−0.7, 0.5)	1.17 (0.6, 1.7)	0.030
Serum total cholesterol (n=8857)	−0.15 (−0.2, −0.1)	−0.19 (−0.2, −0.1)	−0.17 (−0.2, −0.1)	−0.13 (−0.2, −0.1)	−0.09 (−0.2, −0.0)	−0.03 (–0.1, 0.0)	0.001
HDL cholesterol (n=8850)	−0.02 (−0.0, –0.0)	−0.02 (−0.0, –0.0)	−0.02 (−0.0, –0.0)	−0.01 (−0.0, 0.0)	−0.01 (−0.0, 0.0)	−0.01 (−0.0, 0.0)	0.809
Triglycerides (n=8855)	−0.02 (−0.1, 0.0)	−0.04 (−0.1, 0.0)	−0.07 (−0.1, −0.0)	0.00 (−0.1, 0.1)	−0.07 (−0.1, −0.0)	−0.02 (−0.1, 0.0)	0.809
Upper secondary education (n=8567)							
Systolic blood pressure (n=8567)	0.26 (−0.6, 1.1)	0.62 (−0.1, 1.4)	0.31 (−0.3, 0.9)	0.73 (−0.5, 1.9)	0.01 (−0.8, 0.8)	−0.87 (−1.6, −0.1)	0.070
Diastolic blood pressure (n=8567)	0.11 (−0.4, 0.6)	0.02 (−0.5, 0.5)	−0.06 (−0.5, 0.3)	0.63 (−0.2, 1.4)	0.86 (0.3, 1.4)	0.45 (−0.0, 0.9)	0.223
Serum total cholesterol (n=8550)	−0.17 (−0.2, −0.1)	−0.16 (−0.2, −0.1)	−0.20 (−0.2, −0.2)	−0.07 (−0.1, 0.0)	−0.05 (−0.1, 0.0)	−0.02 (−0.1, 0.0)	0.000
HDL Cholesterol (n=8545)	−0.01 (−0.0, 0.0)	−0.00 (−0.0, 0.0)	−0.01 (−0.0, −0,0)	−0.01 (−0.0, 0.0)	−0.00 (−0.0, 0.0)	−0.01 (−0.0, 0.0)	0.815
Triglycerides (n=8550)	−0.04 (−0.1, 0.0)	−0.06 (−0.1, −0.0)	−0.06 (−0.1, −0.0)	0.02 (−0.1, 0.1)	−0.01 (−0.1, 0.0)	0.01 (−0.0, 0.1)	0.207
Short tertiary education (n=4976)							
Systolic blood pressure (n=4975)	1.09 (−0.2, 2.3)	0.91 (−0.1, 1.9)	−0.54 (−1.4, 0.3)	1.21 (−0.3, 2.8)	−1.10 (−2.1, −0.1)	−0.35 (−1.2, 0.5)	0.092
Diastolic blood pressure (n=4975)	0.38 (−0.4, 1.2)	0.08 (−0.6, 0.7)	−0.74 (−1.3, −0.2)	−0.49 (–1.5, 0.5)	−0.08 (−0.8, 0.6)	0.28 (−0.3, 0.8)	0.966
Serum total cholesterol (n=4976)	−0.15 (−0.2, −0.1)	−0.16 (−0.2, –0.1)	0.18 (−0.2, −0.1)	−0.09 (−0.2, 0.0)	−0.04 (−0.1, 0.0)	−0.04 (−0.1, 0.0)	0.030
HDL cholesterol (n=4971)	0.01 (−0.0, 0.0)	−0.01 (−0.0, 0.0)	−0.01 (−0.0, 0.0)	0.00 (−0.0, 0.0)	−0.00 (−0.0, 0.0)	−0.01 (−0.0, 0.0)	0.456
Triglycerides (n=4975)	−0.05 (−0.1, 0.0)	−0.06 (−0.1, 0.0)	−0.12 (−0.2, −0.1)	−0.03 (−0.1, 0.1)	0.00 (−0.1, 0.1)	−0.04 (−0.1, 0.0)	0.782
Long tertiary education (n=5975)							
Systolic blood pressure (n=5975)	0.23 (−0.9, 1.4)	0.15 (−0.8, 1.1)	0.34 (−0.5, 1.1)	−0.62 (−2.1, 0.9)	−1.43 (−2.5, −0.4)	0.13 (−0.6, 0.9)	0.576
Diastolic blood pressure (n=5975)	0.12 (−0.6, 0.9)	−0.24 (−0.8, 0.4)	−0.19 (−0.7, 0.3)	−0.60 (−1.6, 0.4)	−0.23 (−0.9, 0.4)	0.25 (−0.2, 0.7)	0.881
Serum total cholesterol (n=5956)	−0.20 (−0.3, −0.1)	−0.16 (−0.2, −0.1)	−0.14 (−2.0, –0.1)	−0.07 (−0.2, 0.0)	−0.07 (−0.1, –0.0)	−0.04 (−0.1, 0.0)	0.000
HDL cholesterol (n=5955)	0.01 (−0.0, 0.0)	−0.01 (−0.0, 0.0)	0.01 (−0.0, 0.0)	−0.02 (−0.1, 0.0)	−0.02 (−0.0, 0.0)	0.01 (−0.0, 0.0)	0.973
Triglycerides (n=5956)	−0.10 (−0.2, −0.0)	−0.06 (−0.1, 0.0)	−0.06 (−0.1, −0.0)	−0.04 (−0.1, 0.1)	−0.04 (−0.1, 0.0)	−0.06 (−0.1, −0.0)	0.402

Linear mixed models adjusted for age, (n): number of observations pr cardiovascular risk factor, primary education=primary school and secondary school/up to 9 years of education. Upper secondary education=upper secondary school/10–12 years of education. Short tertiary education=university/college <4 years. Long tertiary education=university/college ≥4 years. P value=difference in regression coefficients between Tromsø2 and Tromsø7.

HDLhigh-density lipoprotein

We observed no associations between body height and systolic blood pressure in Tromsø2. Significant inverse associations were observed in Tromsø5–6 in primary or short tertiary educated men. In Tromsø7, there were no associations between body height and systolic blood pressure, except in men with upper secondary education (−0.87 (95% CI –1.6, –0.1). The regression coefficients showed no associations between body height and diastolic blood pressure at most time points. However, we observed significant direct associations between body height and diastolic blood pressure in men with upper secondary education in Tromsø6 (0.86 (95% CI 0.3, 1.4) and primary education in Tromsø7 (1.17 (95% CI 0.6, 1.7)). We observed significant inverse associations in Tromsø2–4 across all educational levels for serum total cholesterol. However, this association attenuated across survey time points and there were no associations in Tromsø7.

There were no associations between body height and HDL cholesterol, apart from a small significant inverse association observed in the primary educated in Tromsø2 (−0.02 (95% CI −0.0, –0.0)). There were no associations between body height and triglyceride levels in the education groups at most survey time points. However, we did observe small significant inverse associations in all education groups in Tromsø4 (primary education: −0.07 (95% CI −0.1, –0.0), upper secondary education: −0.06 (95% CI −0.1, –0.0), short tertiary education: −0.12 (95% CI −0.2, –0.1) and long tertiary education: −0.06 (95% CI −0.1, –0.0).

#### Body height and cardiovascular risk factors at different survey time points

Likelihood-ratio tests showed significant interactions between body height and survey time points (p≤0.03 in all tests except for HDL cholesterol in men (p=0.86)). There were no significant tests of interaction between body height and education. Regression coefficients for the association between body height and cardiovascular risk factors estimated by survey and overall, as the main effect is presented in table 4.

**Table 4:** Linear mixed model regression coefficients (95% CI) for the association between body height and cardiovascular risk factors per 1 SD body height by survey in women and men

	Tromsø21979–1980	Tromsø31986–1987	Tromsø41994–1995	Tromsø52001	Tromsø62007–2008	Tromsø72015–2016	Overall
Women, (n=31 150)							
Systolic blood pressure (n=31 150)	−0.01 (−0.5, 0.4)	−0.25 (−0.7, 0.2)	−0.68 (−1.1, −0.3)	−0.85 (−1.4, −0.3)	−1.62 (−2.1, −1.2)	−1.60 (−2.0, −1.2)	−0.88 (−1.1, −0.6)
Diastolic blood pressure (n=31 150)	−0.31 (−0.6, −0.1)	−0.81 (−1.1, −0.6)	−0.91 (−1.1, −0.7)	−0.74 (−1.1, −0.4)	0.11 (−0.2, 0.4)	0.05 (−0.2, 0.3)	−0.41 (−0.6, −0.3)
Serum total cholesterol (n=31 077)	−0.19 (−0.2, −0.1)	−0.21 (−0.2, −0.2)	−0.21 (−0.2, −0.2)	−0.06 (−0.1, −0.0)	−0.01 (−0.0, 0.0)	−0.01 (−0.0, 0.0)	−0.12 (−0.1, −0.1)
HDL cholesterol (n=31 060)	0.01 (−0.0, 0.0)	0.00 (−0.0, 0.0)	0.01 (0.0, 0.0)	0.02 (0.0, 0.0)	0.03 (0.0, 0.0)	0.04 (0.0, 0.0)	0.02 (0.0, 0.0)
Triglycerides (n=31 075)	−0.05 (−0.1, −0.0)	−0.06 (−0.1, −0.0)	−0.08 (−0.1, −0.0)	−0.05 (−0.1, −0.0)	−0.05 (−0.1, −0.0)	−0.08 (−0.1, −0.0)	−0.06 (−0.1, −0.1)
Men,(n=29 693)							
Systolic blood pressure (n=29 693)	0.60 (0.2, 1.0)	0.66 (0.3, 1.1)	−0.13 (−0.5, 0.2)	−0.22 (−0.8, 0.4)	−1.13 (−1.6, −0.7)	−0.62 (−1.0, −0.2)	−0.10 (−0.3, 0.1)
Diastolic blood pressure (n=29 693)	0.17 (−0.1, 0.4)	−0.19 (−0.4, 0.1)	−0.37 (−0.6, −0.1)	−0.28 (−0.7, 0.1)	0.21 (−0.1, 0.5)	0.57 (0.3, 0.8)	0.05 (−0.1, 0.2)
Serum total cholesterol (n=29 646)	−0.21 (−0.2, −0.2)	−0.22 (−0.3, −0.2)	−0.21 (−0.2, −0.2)	−0.11 (−0.2, −0.1)	−0.06 (−0.1, −0.0)	−0.01 (−0.0, 0.0)	−0.14 (−0.2, −0.1)
HDL cholesterol (n=29 626)	−0.01 (−0.0, 0.0)	−0.01 (−0.0, 0.0)	−0.01 (−0.1, –0.0)	−0.01 (−0.1, 0.0)	−0.01 (−0.1, 0.0)	0.00 (−0.0, 0.0)	−0.01 (−0.0, 0.0)
Triglycerides (n=29 643)	−0.07 (−0.1, −0.0)	−0.08 (−0.1, −0.0)	−0.09 (−0.1, −0.0)	−0.01 (−0.1, 0.0)	−0.04 (−0.1, −0.0)	−0.03 (−0.1, −0.0)	−0.06 (−0.1, −0.0)

Linear mixed models adjusted for age. Overall effect model is adjusted for age and survey time point, (n): number of observations pr cardiovascular risk factor.

HDLhigh-density lipoprotein

The regression coefficients for risk factors varied at each survey time point and there was no clear pattern in the associations, much like our findings presented in tables2  3. In the overall effect model (table 4), we observed a significant inverse association between body height and systolic blood pressure in women (−0.88 (95% CI −1.1, –0.6)). There was no association between body height and systolic blood pressure in men. Likewise, there was an inverse association between body height and diastolic blood pressure in women (−0.41 (95% CI −0.6, –0.3)), while no association was found between body height and diastolic blood pressure in men.

In both sexes, we observed inverse associations in serum total cholesterol (women: −0.12 (95% CI −0.1, –0.1), men: −0.14 (95% CI −0.2, –0.1)) and triglycerides (women: −0.06 (95% CI −0.1, –0.0), men: −0.06 (95% CI −0.1, –0.0)). Additionally, a small direct association between body height and HDL cholesterol in women (0.02 (0.0, 0.0)), and a small inverse association was observed in men (−0.01 (95% CI −0.1, –0.0)).

## Discussion

### Principal findings

The main finding of our study was the inverse associations between body height and all cardiovascular risk factors except for HDL cholesterol in the overall effect models in women. Overall models also showed inverse associations for serum total cholesterol and triglycerides in men. We found no differences in associations between body height and cardiovascular risk factors by survey and educational level. Associations between body height and cardiovascular risk factors varied by survey.

### Body height and cardiovascular risk factors in women

Our finding of inverse associations between body height and blood pressure variables in women are in line with previous cohort studies from Finland and the UK.^25 33^ The inverse associations between body height and systolic and diastolic blood pressure may be interpreted as taller height in women being an advantage in relation to blood pressure, which is consistent with previous studies from Brazil and the UK.^18^,^20^ We found an inverse association between body height and serum total cholesterol in women, which is in line with studies from the UK.^23 34^ These studies reported no association between body height and HDL cholesterol in women, which is contrasted by the small direct association observed in the overall effect model in our study. However, direct associations between body height and HDL cholesterol have previously been found in a 2022 Mendelian randomisation study using data from British and Chinese populations.^35^ In our study, body height was inversely associated with triglyceride levels in the overall effect model. Inverse associations have previously been reported in genetic and Mendelian randomisation studies.^4 6^

### Body height and cardiovascular risk factors in men

Overall effect models showed no association between body height and blood pressure variables, which contrasts with the inverse associations between body height and systolic blood pressure and diastolic blood pressure reported in the UK.^33^ The overall effect models showed no association between body height and diastolic blood pressure in men, which contrasts the direct association previously reported in Scottish and American men.^23 36^ We observed inverse associations between body height and serum total cholesterol, similar to previous findings in Swedish men.^24^ We observed no association between body height and HDL cholesterol in our overall effect analysis, which is in line with previous studies from the UK and Sweden.^23 24 34^ The inverse associations between body height and triglycerides in men in the overall effect model correspond to inverse associations reported in previous genetic and Mendelian randomisation studies.^4 6^

### Social factors, body height and cardiovascular risk factors

Poor early years living conditions, in combination with genetic factors are known to influence body height, attained educational levels and cardiovascular risk factors.^37^,^39^ Mean body height in lower educational groups was lower than in higher educational groups, although the height gap has been reduced in the later Tromsø surveys.^12^ It is possible to speculate that 1 SD taller height be more beneficial regarding systolic blood pressure in the lower education groups. This may also explain why we observed as significant inverse associations in the overall effect model in women and why we observed more significant associations between body height and systolic blood pressure in participants without tertiary education in Tromsø7. Mean serum total cholesterol was reduced across surveys, which might explain why we found no association between body height and serum total cholesterol in Tromsø7.

The shift in the distribution of observations by educational levels over time may also explain why we observed more significant associations with primary and upper secondary education groups and fewer associations with tertiary education groups in Tromsø7.

### Physiological mechanisms, body height and cardiovascular risk factors

The observational design of our study cannot explain the pathophysiological mechanisms between body height and cardiovascular risk factors. However, a 2021 review by Cochrane *et al* suggests that arterial length and dimensions are the main mechanisms behind the inverse associations between body height and systolic blood pressure, with smaller blood vessel dimensions leading to increased arterial pressure and increased systolic blood pressure in shorter individuals.^17^ Shortened return times for reflected pressure waves during the systolic phase in shorter individuals as compared with taller individuals is also a suggested explanation.^40^ The direct associations between body height and diastolic blood pressure might be attributed to higher mean diastolic blood pressure in taller individuals compared with shorter individuals.^41^ This corresponds to our observation of a significant direct association between body height and diastolic blood pressure in men in Tromsø7. Increased bone marrow activity in taller adults compared with shorter adults may mitigate the effect of ageing on the cardiovascular system and reduce arteriosclerosis, a condition related to unfavourable blood pressure and lipid levels.^42^

### Strengths and limitations

To our knowledge, this is the first study to investigate the associations between body height and cardiovascular risk factors at several time points in a Norwegian population.

The main strength of our study is the comprehensive data from The Tromsø Study with measured body height and repeated measurements of blood pressure and lipid levels in surveys conducted over four decades. Specifically, the investigation of associations between body height and lipids in women is important, since most previous studies on body height and lipids focused on men.

Our study has several limitations. Blood pressure measurement methods changed from manual measurements in Tromsø2 to automatic measurements in Tromsø3–7, potentially affecting the validity of comparisons between surveys. However, both manual and automatic measurements have been validated previously in a Norwegian population, finding no differences in systolic blood pressure and diastolic blood pressure in participants with high blood pressure.^43^ Consequently, we decided to use blood pressure data from both manual and automatic measurements in our study.

The variation in units of measurements for body height and educational levels between the Tromsø surveys may be considered limitations of our study. However, checking the data from participants with repeated measures of height revealed a discrepancy of less than 1 cm between height measurements in Tromsø2–4 and Tromsø5–7. Larger differences between measurements in height (3–4 cm) were only observed in participants with more than four survey attendances. These differences are also consistent with expected height loss associated with ageing.^31^ Overall, this supports our decision to use body height data from all surveys in our study since the difference between measurement reporting method was negligible.

The reporting of educational levels varied across The Tromsø Study surveys. Our transformation of years of education reported in Tromsø2–3 and Tromsø5 into educational levels might have led to the loss of information on the effect of each year of additional education. However, the categorisation into educational levels was performed to align with educational levels reported in Tromsø7, allowing us to have one standardised educational level variable in our study.

The results from the linear mixed models may be influenced by multiple testing increasing the likelihood of type I errors. However, adjusting for multiple testing would increase the likelihood of type II errors. Therefore, we chose not to conduct any corrections or adjustments for multiple testing.

We lacked data on parental SES as a potential confounder, which may influence early year living conditions and attained educational levels in offspring, while also being associated with body height. This variable was first introduced in Tromsø6. The family financial situation during childhood may serve as a proxy for early childhood living conditions influencing body height and cardiovascular risk factors.^37 38^ The Tromsø Study contains questionnaire data on the family financial situation during childhood, however, these data were not collected in Tromsø3 and Tromsø5. Therefore, we chose not to include these variables in our study.

## Conclusion

In our study, body height was inversely associated with systolic and diastolic blood pressure in women, and serum total cholesterol and triglycerides in both sexes. These findings are consistent with previous studies from other European countries. Our study showed varied degrees of associations between body height and cardiovascular risk factors at different time points in Norway. Overall, there were no significant associations between body height and cardiovascular risk factors by survey and education. Body height is an established proxy of early year living conditions, which is also associated with an increased risk of cardiovascular disease in adulthood. Our finding of inverse associations between body height and several cardiovascular risk factors may thus be important for policymakers and public health officials working with social inequality in health in Norway.

## supplementary material

10.1136/bmjopen-2024-084109 online supplemental file 1

## Data Availability

Data may be obtained from a third party and are not publicly available.
