# Supplementary figures and images for: Associations between body height and cardiovascular risk factors in women and men: a population-based longitudinal study based on The Tromsø Study 1979–2016
Source: BMJ Open. 2024 Oct 17;14(10):e084109. doi: 10.1136/bmjopen-2024-084109 (PMC11487855; doi:10.1136/bmjopen-2024-084109)

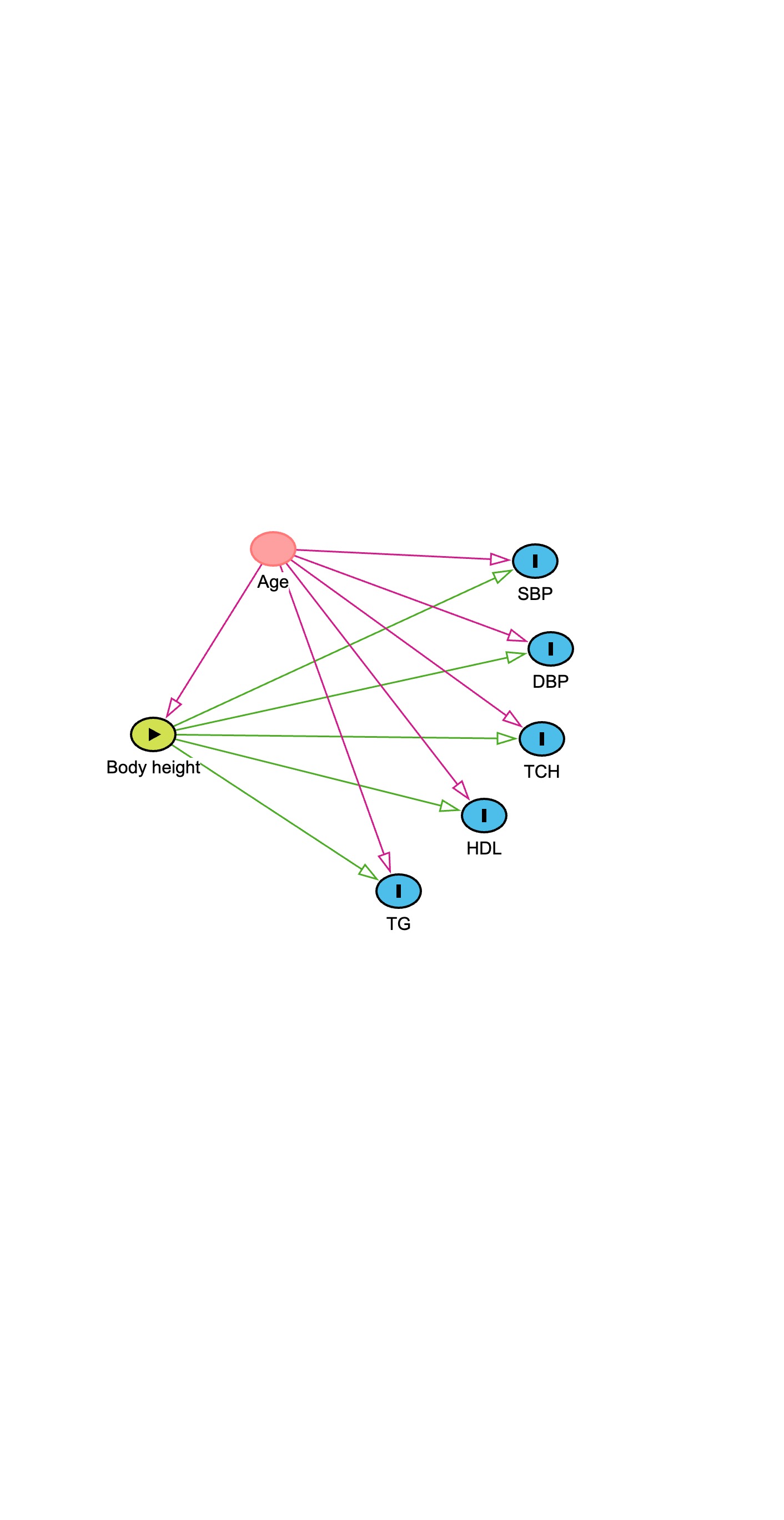

Supplement: online supplemental file 1 [file bmjopen-14-10-s001.jpg]
