# Supplementary material for: Associations between body height and cardiovascular risk factors in women and men: a population-based longitudinal study based on The Tromsø Study 1979–2016
Source: BMJ Open. 2024 Oct 17;14(10):e084109. doi: 10.1136/bmjopen-2024-084109 (PMC11487855; doi:10.1136/bmjopen-2024-084109)
Supplement: Supplementary file 2 [file bmjopen-2024-084109.reviewer_comments.pdf]

PEER REVIEW HISTORY

BMJ Open publishes all reviews undertaken for accepted manuscripts. Reviewers are asked to complete a checklist review form and are provided with free text boxes to elaborate on their assessment. These free text comments are reproduced below.

ARTICLE DETAILS

Title (Provisional)

Associations between body height and cardiovascular risk factors in women and men. A population-based longitudinal study based on The Tromsø Study 1979-2016

Authors

Arntsen, Sondre Haakonson; Wilsgaard, Tom; Borch, Kristin Benjaminsen; Njolstad, Inger; Hansen, Anne Helen

---

VERSION 1 - REVIEW

Reviewer 1
Name Sichieri, Rosely
Affiliation State University of Rio de Janeiro, Department of Epidemiology, Institute of Social Medicine
Date 05-May-2024
COI none

Design of the study should define the cohort based on the seven surveys, instead of " on the In this observational study we used data from the Tromsø Study, which consists of seven population-based surveys conducted between 1974 and 2016. The cohort is described elsewhere, but should be clear in the methods. To further confuse the issue, whether is a panel (analysis of several surveys) or a longitudinal analysis, in line 181-183 each survey is analyzed "Linear regression coefficients were reported per 1 standard deviation (SD) increase in body height and was estimated for each survey"

Line 186-188: "Adjustments for age and anti-hypertensive or lipid lowering medication were based on a Directed Acyclic Graph (DAG) on the pathway between body height and individual cardiovascular risk factors (supplemental figure S1), using DAGitty 32. " Age is ok, classical confounding, but medication is not. Also, in the DAG it is not a confounding.

There are 240 comparisons in tables 2 and 3, how could the few ones statistically significant be valorized?? Also, the analysis related to the p-value in the table's comparison of surveys 2 and 6 is not explained in methods. Is it an expected change ?

Association with cholesterol decrease with time (surveys) and increase for SBP in the primary education? Why? Aging is not an explanation since is age adjusted.

It is not clear why a longitudinal analysis is more informative than the surveys, given the analysis presented.

VERSION 1 - AUTHOR RESPONSE

We thank the editor and the reviewers for the opportunity to submit a major revision of our manuscript.

A document with the reviewer comments and our responses has been uploaded.

Kind regards

Sondre H. Arntsen

Reviewer: 1
Dr. Rosely Sichieri, State University of Rio de Janeiro

Comments to the Author:

Design of the study should define the cohort based on the seven surveys, instead of "on the In this observational study we used data from the Tromsø Study, which consists of seven population-based surveys conducted between 1974 and 2016. The cohort is described elsewhere but should be clear in the methods. To further confuse the issue, whether is a panel (analysis of several surveys) or a longitudinal analysis, in line 181-183 each survey is analyzed "Linear regression coefficients were reported per 1 standard deviation (SD) increase in body height and was estimated for each survey"

Thank you for this comment. The population-based Tromsø Study consists of seven surveys, where inhabitants in the Tromsø municipality are invited to attend again if they are still living in the municipality. This allowed us to have longitudinal study design with staggered entries for the participants in our study. We have added a few sentences about this in the design and data section of the methods (lines 116-122, 125-132). We have made changes to the stated study design in the abstract (line 36).

We agree that the wording in the previous version of the submitted manuscript may have been confusing on whether the presented analyses was panel analyses or longitudinal analyses. In the revised manuscript, the analyses presented in table 2-4 is longitudinal analyses of several survey time-points, where 60.4% of our participant contribute with two or more measurements for cardiovascular risk factors (lines 153-157). The line *"Linear regression*

coefficients were reported per 1 standard deviation (SD) increase in body height and was estimated for each survey" was an attempt at stating that each regression coefficient reported in table 2 and 3 was a regression coefficient estimated for each survey based on linear mixed models. Based on your comments and the comments from reviewer 2, we have made changes the description of the linear mixed models in the manuscript (lines 217-223).

Line 186-188: "Adjustments for age and anti-hypertensive or lipid lowering medication were based on a Directed Acyclic Graph (DAG) on the pathway between body height and individual cardiovascular risk factors (supplemental figure S1), using DAGitty 32. "Age is ok, classical confounding, but medication is not. Also, in the DAG it is not a confounding.

Thank you for pointing this out. Medication status in the submitted DAG was a mediator on the pathway between height and each CVD risk factor and should not be adjusted for in the analyses. We have made amendments to the analyses, so that the results presented in table 2 and table 3 are adjusted for age only. We have revised the statement about adjustment variables (line 163-164) and uploaded a revised DAG with age as the only adjustment variable for our main analyses. Information on medication status have been removed from the manuscript since it is no longer used in our study.
There are 240 comparisons in tables 2 and 3, how could the few ones statistically significant be valorized?? Also, the analysis related to the p-value in the table's comparison of surveys 2 and 6 is not explained in methods. Is it an expected change?

For better readability of our manuscript, we made the decision to valorize the few statistically significant regression coefficients. However, we do also point out that at we found no associations between height and CVD risk factors at several time-points in our study (lines 270-272 and 304-306). We have added a sentence clarifying the p-value and its use in the revised manuscript (lines 240-244, 249). Regarding the question of whether it was an expected change. We find that change was as expected based on previously reported changes in mean body height and CVD risk factors by education in the period between Tromsø2 and Tromsø7.

Association with cholesterol decrease with time (surveys) and increase for SBP in the primary education? Why? Aging is not an explanation since is age adjusted.

Thank you for this comment. We agree that ageing is not an explanation since the LMMs are age-adjusted. Mean serum total cholesterol was reduced in the later surveys (Tromsø5-6) compared to Tromsø2, which may partly explain why the association decrease with survey (see Table 1). We have added a sentence about this in the manuscript (lines 411-412). The changes in the associations for serum total cholesterol and SBP in participants with primary education may also be the result of a calendar time-effect with changes in the composition of education groups in the different surveys with a larger percentage of observations in primary education in the early surveys compared to the later surveys (see table 1 in the manuscript and lines, 265-266). This is also consistent with findings from a previous study on trends in CVD risk factors using The Tromsø Study (https://pubmed.ncbi.nlm.nih.gov/24675289/).

Another possible explanation is the changes in mean height in each education group with the addition of new participants in each survey. Previous work by our group showed that mean height in women and men with low education increased in the period between Tromsø2 and Tromsø7. Thus, the impact of 1SD taller height on the associations between height and CVD risk factors might be reduced if participant mean height is taller in the later surveys of The Tromsø Study (https://journals.plos.org/plosone/article?id=10.1371/journal.pone.0279965). We have added a sentence about this in the manuscript (lines 403-409).

It is not clear why a longitudinal analysis is more informative than the surveys, given the analysis presented.

Based on the previous version of the manuscript, we are inclined to agree that it was not clear why longitudinal analyses would be more informative than the surveys. However, in the revised version of the manuscript, we use all data with repeated measurements in one linear mixed model per CVD risk factor with interaction terms for education and survey time-point. This allowed us to estimate and compare associations between height and CVD risk factors. We would thus argue that longitudinal analyses have clear advantages compared to surveys.
Reviewer: 2
Dr. Mohammed Moinuddin, Edge Hill University
Please also see attached document.

Comments to the Author:
The study aimed to investigate the association between body height and cardiovascular risk factors over time in men and women across the educational levels in Norway. An interesting topic and the authors used a large dataset with repeated measures on several outcome variables to serve the investigation. The analytical approach and presentation of the outputs need a significant change to enhance readability and draw valid conclusions.

We are grateful that reviewer 2 found the topic of our manuscript interesting and for his thorough feedback and suggestions on how to best improve our manuscript. Our responses are provided below.

Comments and feedback
1. Inconsistent measurement tools. For measuring the outcome variables blood pressure, serum total cholesterol, HDL cholesterol, and triglyceride concentrations different approaches are adopted at different time points. The independent variable body height was also measured at different precision levels at different time points. No conversions were used to make the measurements comparable or performed any checks to ensure that the variables captured at different time points are giving similar information. Therefore, it is difficult to justify the validity of the comparison over time.

Thank you for pointing this out. We have added a section about the different BP measurements to the limitations section of our manuscript (lines 445-451). On the other hand,

we would argue that our use of repeated measurements for our dependent variables and the new implementation of survey time-point as a covariate in our mixed models allowed us to adjust for mean changes in systolic and diastolic blood pressure related to changes in measurement methods. A Norwegian validation study on the use of manual and automatic blood pressure measurements comparable to our measurement methods was conducted in 1997 (https://www.ntnu.no/ojs/index.php/norepid/article/view/413). This study found no differences in systolic blood pressure between manual and automatic measurements. The automatic measurement method provided slightly lower diastolic blood pressure values than manual measurement. However, the between methods differences diminished with higher diastolic blood pressure. Thus, we would argue that our use of manual and automatic BP measurements without conversions is justified in the context of our study.

The analyses methods used for the lipid variables were similar across the surveys and the analyses were conducted within 48 hours at the same hospital. It is possible that the use of non-fasting blood samples in our study might lead to slightly increased lipid levels compared to lipid levels taken from fasting blood samples.

On the other hand, a 2016 joint statement from the European Atherosclerosis Society and European Federation of Clinical Chemistry and Laboratory Medicine (https://pubmed.ncbi.nlm.nih.gov/27122601/) stated that the differences in measured lipid levels between sampling methods were minor and that the use of non-fasting blood samples is acceptable. We are therefore confident that our use of non-fasting blood samples collected at different time-points is justified.

We have added a section about the different height measurements to the limitations section of our manuscript (453-460). In our study, we have used height measurements at first attendance as our independent variable. However, our dataset also contains height measurements for participants with more than one attendance. Following your comment, we have checked height measurements for our study participants again, finding a within 1 cm agreement between the height reported to the nearest 1 cm as in Tromsø2-4 and height reported to nearest 0.1 cm in Tromsø 5-7. We have added two sentences about this in the variable section of the manuscript (lines 191-194). Larger height differences between surveys related to height loss were only observed in participants with several attendances and followed the pattern of height loss expected with ageing. Since we only use height at first participant attendance, we find that the use of height measurements from all six surveys of The Tromsø Study without performing any conversions is justified in our study.

2. Main assumptions. Mean (SD) and linear models were applied for all the outcome variables. No justification based on their distribution pattern was presented. Also, the linear model needs to satisfy the linearity assumption. I would advise checking the data distribution, checking if there are any outliers and the association pattern between outcomes and exposure. Based on these checks fit the appropriate models.

We thank reviewer 2 for this comment. We have checked the distribution and assumption patterns again and found that they were adequate. We have fitted the appropriate models for the revised study and made amendments to the statistical analyses section of the manuscript (lines 217-223).

3. LMM specification. The authors used a linear mixed model which is the right choice for the data structure. However, what correlation structure they used is not clear. Also, it is mentioned that the adjustment for available confounders was based on DAG. I wonder how the DAG-based associations are implemented in the model. It would be great to see the model specification in the manuscript. So that the reader can understand and interpret the results correctly. The beta coefficients are interpreted as 'for one SD change in body height'. How was the independent variable body height scaled in the model?

Thank you for this comment and helpful suggestion. We have made amendments to the statistical analyses section of the manuscript (lines 217-227). The scaling for one standard deviation body height in the LMM was 6.2 cm in women and 6.7 cm in men (line 223).

4. Interaction terms. It seems the authors estimated the models in subgroups stratified by sex, education, and surveys. This means there would be many models for each outcome variable. This makes it difficult to compare estimates even at different subgroups. Instead, the authors can run a single model for each outcome fitting with the interaction terms included. This will allow comparing the estimates and drawing inferences about the difference between the parameters.

Thank you for this helpful comment and suggestion. In our revised analyses, we have fitted single models with main effects of height and indicator variables of survey time point and education and with all two-way and three-way cross product terms between the variables Following Reviewer 1s comments about medication being a mediator om the pathway between age and CVD risk factors, we have changed our main models to include age as the only adjustment variable in our main analyses (lines 163-164 and table 2-3)

5. P-value. A single column of p-value is reported in Tables 2 and 3. What was the hypothesis tested here and what statistical test was used? Was any correction (for example Bonferroni) used to adjust for multiple testing?

Thank you for this comment. The single column of p-value in table 2 and table 3 was taken from the linear mixed model and used to assess if the was a significant difference in regression coefficients at the first and last survey time-point. We agree that it was not adequately reported in the methods section of the previous version of the manuscript, and we have added two sentences about our use of Wald tests and p-values for in the revised version (lines 238-242, line 247).

A section about multiple testing have been added to the limitations of our study (lines 468-471). We did not conduct any correction to adjust for multiple testing since the implementation of a Bonferroni test for multiple testing would have been to conservative, increasing the likelihood of type II errors. However, if we had adjusted for multiple testing by adjusting by a factor of five since we have five models pr sex. We would have gone from six to three significant tests in women (table 2) and from three to zero in men (table 3).

6. Conclusion. A strong conclusion was made (for example in lines 216-17) based on the results. However, can these results be due to random chance? Among hundreds of tests, only a very few found significant based on p-values.

Thank you for this comment. We agree with you that the previous version of the manuscript had some strong statements regarding the reported findings, which may have been due to random chance. The text in revised version of the manuscript has been changed to tone down impact of the regression coefficients presented in tables 2-3 and generally contain less statements about our findings based on single time-point regression coefficients.
Regarding the statement in lines 216-217 in the previous version of the manuscript was an attempt at summarizing the descriptive characteristics presented in table 1.

7. Missing values and outliers. I wonder how the authors handled the missing values or any outliers in the data set. Please describe.

We acknowledge that missing values and outliers should have been described better in the previous version of the manuscript. Before we did our analyses, we conducted summary statistics on height and each CVD risk factor variables to check for outliers, excluding participants with missing or invalid height measurements or missing CVD risk factor measurements as stated in lines 149-151 in the revised manuscript. The main advantage of our use of linear mixed models is the that it allows for the inclusions of participants with missing values if they have at least one measurement for the dependent variables. Since our analyses contains more than 60 000 observations of which several are repeated measures, we are confident that the presence of outliers did not alter our findings. We therefore decided to keep the outliers in the presented analyses in tables 2-4.

8. Use graphs. The manuscript looks very data dense. Using graphs (heatmaps or point-range plots) can increase the readability.

Thank you for this comment. While we agree that the implementation of heat-maps in lieu of table 2 and table 3 may have increased the readability of our manuscript. We find that the addition of tests for interaction between height, survey and education, the newly added table 4 with regression coefficients for overall main effect and the general rewriting of the manuscript has adequately increased its overall readability.

9. Line 131, 133. Both numbers are 333, is it a coincidence or a typo?

Thank you for pointing this out. We have checked the data again and found that we excluded 333 participants who withdrew consent to participate in The Tromsø Study. We also excluded 333 participants with invalid of missing height at first attendance. Thus, the numbers reported at line 131 (line 148 in the revised manuscript) and 133 (line 150 in the revised manuscript) both being 333 is merely a coincidence.

However, when we revised our paper, we did find a bad error in the reported number of participants in the previous version of the manuscript. The correct number of participants in our study is 23 512 participants, not 31 470 participants as stated in previous version of the manuscript. However, this should not have any impact on our reported findings in the revised manuscript since we have done new analyses for table 2-4 and table 1 report findings by number of observations only. We have revised the abstract (line 44), manuscript (line 152) and figure 1 to contain the correct number of participants.

Reviewer: 1
Competing interests of Reviewer: none

Reviewer: 2
Competing interests of Reviewer: I have no competing interests.

Thank you again to both reviewers for your thorough review, which was most helpful when revising our manuscript. We hope that our revisions and comments will meet the reviewers and editor's concerns and that our manuscript will be considered acceptable for publication.

Kind regards

Sondre Haakonson Arntsen

Corresponding author

UiT The Arctic University of Norway

VERSION 2 - REVIEW

Reviewer **1**

Name **Sichieri, Rosely**

Affiliation **State University of Rio de Janeiro, Department of Epidemiology, Institute of Social Medicine**

Date 26-Sep-2024

COI none

All questions raised were answered

Reviewer 2

Name Moinuddin, Mohammed

Affiliation University of Central Lancashire, School of Medicine and Dentistry

Date 03-Oct-2024

COI I have no competing interests.

I thank the authors for their efforts in making all the data processing and statistical analysis approaches clearer with the justification provided. The current version is a much clearer reading to me. Thanks and congratulations.
